# Type B Lactic Acidosis Secondary to Malignancy: Case Report, Review of Published Cases, Insights into Pathogenesis, and Prospects for Therapy

**DOI:** 10.1100/tsw.2011.125

**Published:** 2011-07-07

**Authors:** Juan P. Ruiz, Ashok Singh, Peter Hart

**Affiliations:** ^1^ Department of Medicine, John H. Stroger Hospital of Cook County, Chicago, Illinois, USA; ^2^ Division of Nephrology, John H. Stroger Hospital of Cook County, Chicago, Illinois, USA

**Keywords:** lactic acidosis, neoplasm, pathogenesis, treatment

## Abstract

Most of the information about type B lactic acidosis associated with cancer is derived from case reports and there are no randomized controlled trials to compare different therapeutic modalities. Previous reviews of cases only refer to hematologic malignancies. We present a patient with non-Hodgkin's lymphoma who developed type B lactic acidosis. We performed a search of the PUBMED database using the MESH terms “neoplasms” AND “acidosis, lactic”, limited to the English language, and written between the years 2000 and 2010. A total of 31 cases were retrieved. These cases were identified and reviewed. The possible pathophysiologic mechanisms and treatment options are discussed. Type B lactic acidosis is most commonly seen in patients with lymphoma or leukemia. Although formal prospective trials are lacking, type B lactic acidosis in patients with cancer seems to be a marker of poor prognosis regardless of the treatment offered and may be invariably fatal. Future research should focus on potential therapy based on the pathogenic mechanisms that lead to type B lactic acidosis in cancer patients.

## CASE PRESENTATION

A 55-year-old woman with a history of bronchial asthma and chronic kidney disease (stage 3) of unclear etiology presented to the Emergency Department (ED) of a large urban teaching hospital complaining of 2 months of bilateral lower extremity swelling, progressive shortness of breath, and decreased appetite. She was diagnosed with acute lymphocytic leukemia at another institution, but failed to follow up with her initial hematology appointments. She denied environmental exposures, alcohol or illicit drug use, or tobacco smoking. Her family history was not remarkable and she was not taking any medications.

On admission, the patient was tachycardic (103 beats/min) and her blood pressure was elevated (169/98 mmHg). Her physical exam was remarkable for nonpainful lymphadenopathy of the neck and lower extremity edema. Her laboratory studies (Table 1) showed the following: serum sodium 142 meq/L, potassium 3.5 meq/L, chloride 92 meq/L, bicarbonate ($HCO_3$) 22 meq/L, anion gap 28, blood urea nitrogen

**TABLE 1**
**Patient's Lab Values**

| Hospital Day | 1 | 4 | 5 | 6 | 7 | 8 | 9 | 10 | 11 | 12 | 13 | 14 | 15 | 16 | 17 | 21 |
|---|---|---|---|---|---|---|---|---|---|---|---|---|---|---|---|---|
| pH arterial | NP | NP | 7.174 | 7.218 | 7.263 | 7.062 | 6.816 | 7.491 | 7.348 | 7.249 | 7.259 | 7.247 | 7.267 | 7.321 | NP | 7.489 |
| $HCO_3$ (meq/L) | 22 | 9 | 8 | 20 | 8 | 9 | 4 | 18 | 22 | 25 | 25 | 25 | 23 | 23 | 21 | 19 |
| Anion gap | 28 | 33 | 34 | 17 | 25 | 25 | 37 | 24 | 18 | 13 | 14 | 13 | 16 | 13 | 10 | 13 |
| Lactic acid (mmol/L) | NP | NP | 12.7 | 14.1 | 10.5 | 15 | 19 | 6.1 | 6.0 | 4.0 | 3.1 | 2.4 | 3.4 | 1.8 | NP | 1.5 |
| Creatinine (mg/dL) | 1.8 | 2.3 | 2.6 | 1.1 | 3 | 3.1 | 3.7 | 2.4 | 3.3 | 3.1 | 3.8 | 4.1 | 4.3 | 4.1 | 3.3 | 1.6 |

NP: not performed.

(BUN) 37 mg/dL, creatinine 1.8 mg/dL, and uric acid 10.1 mg/dL. Serum calcium, phosphorus, glucose, and liver function tests were within the normal range. Lactate dehydrogenase (LDH) was slightly increased (258 U/L). Her complete blood count showed pancytopenia (hemoglobin 10.2 g/dL, white blood cells 2.5 k/µL, platelets 115 k/µL). An intravenous contrast-enhanced computed tomography of the chest and abdomen showed retroperitoneal adenopathies, and an ultrasound of the kidneys revealed normal-sized echogenic kidneys.

On the day of admission, the patient was managed with furosemide 40 mg per oral (po) and metoprolol 50 mg po every 12 h for blood pressure control. A transthoracic echocardiogram was unremarkable. On hospital day 5, despite being asymptomatic, the patient became hypotensive and oliguric. A repeat serum creatinine increased to 2.6 mg/dL, arterial lactate was 12.7 mmol/L (0.5–2.2 mmol/L), pH decreased to 7.174 ($PCO_2$ 22.6 mmHg, $HCO_3$ 8 mmol/L), and serum glucose dropped to 39 mg/dL. She was transferred to the Medical Intensive Care Unit (MICU) and was treated with an isotonic saline solution, showing rapid improvement of her blood pressure levels after a few hours. The origin of the acute kidney injury was thought to be a combination of contrast nephropathy and acute tubular necrosis from the episode of transient hypotension. Hemodialysis was initiated due to severe metabolic acidosis. Her lactic acid levels remained elevated despite being hemodynamically stable (mean arterial pressure above 65 mmHg, systolic blood pressure above 90 mmHg) and never showing signs of tissue hypoperfusion, ruling out type A lactic acidosis. On hospital day 8, a diagnosis of type B lactic acidosis was made and thiamine (100 mg intravenously q 12 h) was started. The results of immunostaining of a bone marrow biopsy were compatible with a large B-cell non–Hodgkin's lymphoma. There was a plan to start chemotherapy, but given her abnormal acid base status and kidney function, a decision was made to start only with prednisone 100 mg po daily. On hospital day 9, and after completing four sessions of hemodialysis, her mental status deteriorated and endotracheal intubation was performed for airway protection. Her arterial pH dropped to 6.816, $HCO_3$ was 4 meq/L, and lactate was 19 mmol/L. She was given a bicarbonate drip during the next 2 days and hemodialysis was stopped on day 10. No episodes of hypoxia or hypotension were documented.

On hospital day 11, the patient's mental status improved and she was extubated. Her pH increased to 7.348 ($PCO_2$ 42.1 mmHg, $HCO_3$ 22 meq/L), lactate was 6.0 mmol/L, and it continued to decrease to normal levels during the following week (Fig. 1). The rest of the chemotherapy (cyclophosphamide, doxorubicin, vincristine, and intrathecal methotrexate) was started. The patient, however, became progressively pancytopenic. She developed neutropenic fever, secondary to vancomycin-resistant enterococci and coagulase-negative staphylococci bacteremia, and progressed to refractory septic shock and multiorgan failure. She died on hospital day 30.

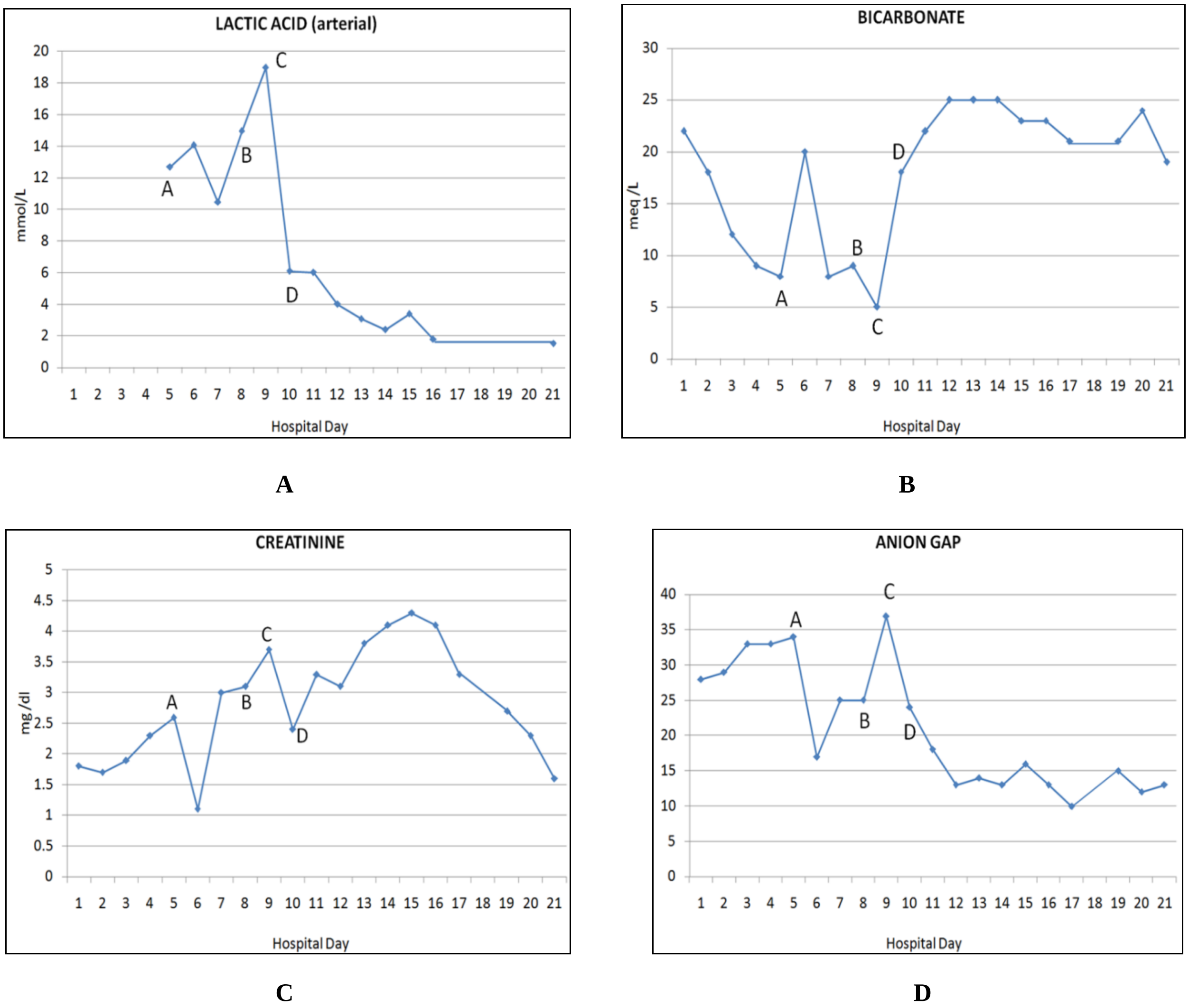

**FIGURE 1.** Variation of lactic acid (A), bicarbonate (B), creatinine (C), and anion gap (D) before the patient became septic. A: transient hypotension, development of acute renal failure, hemodialysis started; B: thiamine and prednisone started; C: bicarbonate drip started; D: hemodialysis and bicarbonate drip discontinued.

## REVIEW OF CASES

We performed a search of the PUBMED database using the MESH terms “neoplasm” AND “acidosis, lactic”, limited to the English language, humans, and published between the years 2000 and 2010. A total of 31 cases were identified and reviewed (see Table 2).

Most of the cases of type B lactic acidosis were associated with hematologic malignancies (87%). Lymphomas were the predominant disorder (58%), of which 16 cases (52%) were secondary to non–Hodgkin’s lymphoma, one case (3%) was secondary to Hodgkin’s lymphoma, one case (3%) was secondary to multiple myeloma, and one case (3%) was presumed to be lymphoma, but could not be classified. Eight cases (26%) were associated with leukemia, five of which (16%) were associated with acute lymphocytic leukemia, two (6%) with acute myeloid leukemia, and one (3%) with chronic lymphocytic leukemia.

**TABLE 2**
**Case Reports of Type B Lactic Acidosis Secondary to Malignancies in the Past 10 Years (2000–2010) and Their Response to Treatment**

| Case [Ref.] | Age | Gender | Diagnosis | Comorbidities | LA Pretreatment | Hypoglycemia | Liver Involvement | Treatment Received | LA Post-Treatment | Outcome |
|---|---|---|---|---|---|---|---|---|---|---|
| 1 [16] | 65 years | Male | Large B-cell lymphoma | Stable CAD | 17 mmol/L | Yes | ASTx5 ULN, ALTx2 ULN | SLED, chemotherapy | 4.3 mmol/L | Favorable |
| 2 [19] | 81 years | Male | Mantle cell lymphoma | DM2, HTN, hypothyroidism | 13.6 mmol/L | Yes | No | Chemotherapy | 4.8 mmol/L | NM |
| 3 [1] | 79 years | Male | Acute myeloid leukemia | Acute lymphoblastic leukemia, HTN | 19 meq/L | Yes | No | Supportive | NM | Fatal (days) |
| 4 [1] | 75 years | Female | Follicular lymphoma | None | 4.8 meq/L | NM | ASTx10 ULN, ALTx5 ULN | Chemotherapy | 5.4 meq/L | Fatal (days) |
| 5 [1] | 54 years | Female | Large B-cell lymphoma | Chronic lymphocytic leukemia | 12 meq/L | No | AST 179 IU/L, ALT 106 IU/L (ULN not mentioned) | Chemotherapy | NM | Fatal (1 month) |
| 6 [1] | 54 years | Male | T-cell lymphoma | None | 12 meq/L | No | No | Chemotherapy | NM | Fatal (10 weeks) |
| 7 [1] | 66 years | Female | Chronic lymphocytic leukemia | None | 5.3 meq/L | No | No | Chemotherapy | 5.3–7.5 meq/L | Fatal (unknown) |
| 8 [1] | 61 years | Female | Presumed lymphoma (not typified) | HTN | 11.6 meq/L | No | No | Chemotherapy | NM | Fatal (days) |
| 9 [1] | 54 years | Male | Diffuse large B-cell lymphoma | ESRD | 16.9 meq/L | Yes | No | Supportive | NM | Fatal (days) |
| 10 [20] | 74 years | Male | Burkitt's lymphoma | NM | 15.8 mmol/L | Yes | ASTx2 ULN, ALT normal | Supportive | NM | Fatal (days) |
| 11 [13] | 11 months | Female | B-cell lymphoma | NM | 18.6 mmol/L | No | No | Thiamine | 6.7 mmol/L | Favorable |
| 12 [21] | 7 years | Male | Pre–B-cell acute lymphocytic leukemia | None | 8.4 mmol/L | No | No | Chemotherapy | 1 mmol/L | Favorable |
| 13 [17] | 29 years | Male | T-cell acute lymphoblastic leukemia | None | 8 mmol/L | No | AST < x2 ULN, ALTx2 ULN | Chemotherapy | 1 mmol/L | Favorable |
| 14 [22] | 64 years | Female | MALT lymphoma | NM | 9 mmol/L | Yes | NM | Chemotherapy | NM | Fatal (days) |
| 15 [2] | 11 years | Female | T-cell acute lymphoblastic leukemia | None | 10.8 mmol/L | Yes | NM | Chemotherapy, CVVH ($NaHCO_3$ in replacement fluid) | 16.6 mmol/L | Fatal (days) |
| 16 [2] | 17 years | Male | T-cell acute lymphoblastic leukemia | None | 16.0 mmol/L | No | NM | Bicarbonate infusion | NM | Fatal (hours) |
| 17 [2] | 18 years | Female | Large-cell immunoblastic T-cell lymphoma | None | 15.4 mmol/L | Yes | NM | Chemotherapy | NM | Fatal (months) |
| 18 [23] | 20 years | Male | Acute myeloid leukemia | None | 8.2 mmol/L | Yes | NM | Chemotherapy | NM | Fatal (days) |
| 19 [24] | 82 years | Male | Hodgkin's lymphoma | None | 11.5 mmol/L | Yes | NM | Chemotherapy, bicarbonate drip | NM | Fatal (days) |
| 20 [18] | 28 years | Male | NK/T-cell lymphoma (nasal type) | None | 11.2 mmol/L | Yes | Increased liver enzymes (no ULN) | Chemotherapy | 1.05 mmol/L | Fatal (days) |
| 21 [25] | 64 years | Male | Small-cell lung cancer | None | 15.8 (units NM) | No | Extensive nodularity of liver (MRI) | Dialysis | NM | Fatal (days) |
| 22 [26] | 24 years | Male | Immunoblastic lymphoma with AML M3 transformation | None | 12 mmol/L | Yes | Increased liver enzymes (no ULN) | Hemodialysis, chemotherapy | NM | Fatal (days) |
| 23 [27] | 77 years | Male | Mantle cell lymphoma | None | 237 mg/dL (3.0–17) | No | No | Chemotherapy | NM | Favorable |
| 24 [28] | 7 years | Male | Pre–B-cell acute lymphoblastic leukemia | None | 5.5 mmol/L | Yes | No | Thiamine, riboflavin | 30 mmol/L | Fatal (days) |
| 25 [29] | 33 years | Male | Anaplastic large B-cell lymphoma | NM | 11.1 mmol/L | NM | Yes (autopsy) | NM | NM | Fatal (days) |
| 26 [29] | 48 years | Female | Anaplastic large B-cell lymphoma | NM | 6.19 mmol/L | NM | Yes (autopsy) | NM | NM | Fatal (days) |
| 27 [29] | 45 years | Female | Anaplastic large B-cell lymphoma | NM | 6.5 mmol/L | NM | Yes (autopsy) | NM | NM | Fatal (days) |
| 28 [30] | 14 years | Female | Metastatic undifferentiated cancer of unknown primary site | None | 199 mg/dL (ULN NM) | NM | Increased liver enzymes (ULN NM) | NM | NM | Fatal (days) |
| 29 [31] | 25 years | Female | Undifferentiated carcinoma of unknown primary site | None | 171.5 mg/dL (5–15 mg/dL) | No | Hypodense nodularities on CT; ASTx6 ULN, ALT mildly elevated | Hemodialysis, bicarbonate infusion | NM | Fatal (days) |

*Table 2 continues*

**TABLE 2 (continued)**

| Case [Ref.] | Age | Gender | Diagnosis | Comorbidities | LA Pretreatment | Hypoglycemia | Liver Involvement | Treatment Received | LA Post-Treatment | Outcome |
|---|---|---|---|---|---|---|---|---|---|---|
| 30 [32] | 70 years | Male | Cholangiocarcinoma (poorly differentiated) | Ulcerative colitis | 12.5 mmol/L | No | ASTx3 ULN, APx2 ULN; CT: 12- x 8- x 15-cm mass in the right lobe of the liver | Hemodialysis, bicarbonate infusion | 24 mmol/L | Fatal (days) |
| 31 [33] | 55 years | Male | Multiple myeloma | NM | 6.3 mmol/L | No | NM | Chemotherapy | NM | Fatal (1 year) |

ALT, alanine transferase; AML, acute myelocytic leukemia; AP, alkaline phosphatase; AST, aspartate transferase; CAD, coronary artery disease; CT, computed tomography; CVVH, continuous veno venous hemofiltration; DM2, diabetes mellitus type 2; ESRD, end-stage renal disease; HTN, hypertension; LA, lactic acidosis; MRI, magnetic resonance imaging; NM, not mentioned; SLED, sustained low-efficiency dialysis, ULN, upper limit of normal.

Nonhematologic malignancies were described in only four patients (13%). Two cases (6%) were undifferentiated tumors of unknown primary sites, and single cases were secondary to small-cell lung cancer (3%) and poorly differentiated cholangiocarcinoma (3%)

A majority of the cases occurred in adults. Twenty-five patients (81%) were above 18 years of age (16 [52%] were 50 years or older, and nine [29%] were between 18 and 49 years old). Only six cases (19%) occurred in children, mostly due to hematologic malignancies: four cases (13%) of acute lymphocytic leukemia, one case (3%) of non–Hodgkin's lymphoma, and one (3%) case was associated to a metastatic, undifferentiated cancer of unknown primary site.

Liver involvement was described in 14 patients (45%). Ten of these cases (32%) were associated with elevated transaminases, with values of aspartate aminotransferase (AST) ranging between two and ten times the upper limit of normal, and alanine aminotransferase (ALT) levels ranging between two and five times the upper limit of normal. However, in four cases (13%), the upper limit of normal of the reference value was not mentioned. Liver involvement was determined by imaging studies in three cases (10%): in one patient, computed tomography showed hepatomegaly with multiple hypodense nodules involving both hepatic lobes; in another patient, it showed a 12- × 8- × 15-cm mass in the right lobe of the liver; and in the third patient, a magnetic resonance image showed extensive nodularity of the liver. In three patients (10%), liver involvement was established postmortem.

Hypoglycemia was documented in 13 (42%) patients, glucose levels were not mentioned in five cases (16%), and in the remainder (42%), glucose levels were normal.

The treatment modalities used included chemotherapy, dialysis, intravenous thiamine, sodium bicarbonate infusion, and supportive management. Eighteen patients (58%) received chemotherapy and six patients (19%) underwent renal replacement therapy. The specific method of dialysis was only mentioned in two (6%) patients: one had sustained low-efficiency dialysis (SLED) and the other received continuous veno venous hemofiltration (CVVH). Two patients (6%) received thiamine supplementation for presumed thiamine deficiency, even though thiamine activity levels in blood (transketolase activity in red blood cells) were not determined. Five patients (16%) received bicarbonate infusions. However, there was no description of the type of infusions (e.g., isotonic). In three patients (10%), supportive measures were the sole treatment offered.

Twenty-five of the 31 cases (81%) had a fatal outcome. Death occurred in a matter of hours or days from the onset of lactic acidosis in 20 patients (64%) and three (10%) died within 3 months. In one case, the outcome of the patient was not mentioned. Of the five patients (16%) who had a favorable outcome, one (3%) received chemotherapy and SLED, one (3%) received thiamine, and the remaining three (9%) received chemotherapy alone. Also, in the group of those who survived, two patients (40%) were under 18 years old, one patient (20%) was between 18 and 50 years old, and two patients (40%) were above 50 years old.

## PATHOGENESIS

Lactic acid (LA) results from the binding of one molecule of lactate with one hydrogen cation[3]. Lactic acidosis is defined as LA levels in whole blood above 5 mmol/L and a pH less than 7.30[4]. In normal conditions, lactic acid is cleared by the liver (80–90%) after conversion to glucose via gluconeogenesis, and the remainder by the kidneys.

Lactic acidosis has been traditionally classified according to the underlying pathophysiologic process. Type A lactic acidosis is present in the setting of hypoxia and poor tissue perfusion. Type B lactic acidosis occurs in normoxic and normal perfusion states, and is associated with underlying liver disease (decreased clearance of lactate), diabetes mellitus, thiamine deficiency, mitochondrial toxins (e.g., alcohols, salicylates, reverse transcriptase inhibitors[5,6]), seizures, malignancies, or hereditary enzymatic defects, among other causes. Type D lactic acidosis is due to excessive production of D-lactic acid from intestinal bacterial proliferation, such as in patients with short bowel syndrome, ischemic bowel disease, or small bowel obstruction[7] (note: while mammals produce L-lactic acid, bacteria produce D-lactic acid).

To understand the mechanisms involved in the development of type B lactic acidosis in patients with cancer, it is important to review the metabolism of a normal and a malignant cell.

In adequate conditions of oxygenation and nutrient supply, a normal cell obtains its energy requirements from the glycolytic production of pyruvate, followed by the conversion of pyruvate to acetyl CoA by the enzyme pyruvate dehydrogenase, which uses thiamine as a cofactor (Fig. 2). Acetyl CoA then enters the tricarbonic acid cycle (TAC) that takes place in the mitochondria. This process results in the production of 36 molecules of ATP from a single molecule of glucose. In the absence of oxygen, the TAC in the mitochondria cannot occur and therefore the cell has to depend on the glycolytic pathway to derive energy. The glycolytic pathway only supplies four molecules of ATP by virtue of the conversion of accumulated pyruvate to lactic acid[12]. Lactic acid is then recycled back to glucose in the liver by the gluconeogenic process known as the Cori cycle, in which two molecules of lactic acid produce one molecule of glucose. However, as noted by Otto Van Warburg in 1924[8], tumor cells switch their metabolic machinery towards the glycolytic state, even in the presence of normal oxygen concentrations (known as aerobic glycolysis or the Warburg effect). Additionally, because the Cori cycle requires six molecules of ATP to produce a single molecule of glucose and therefore more energy is expended by the cell to regenerate glucose than is generated in converting glucose to lactate, it would seem that the cell would be highly inefficient in terms of energy production. The reason for these metabolic changes in a tumor cell is not known, but as proposed by Vander Heyden et al.[9], it is possible that these changes enable cancer cells to acquire and metabolize nutrients in a way that favors proliferation over efficient ATP production.

It seems, then, that the excessive production of lactic acid in patients with cancer is counterbalanced by the Cori cycle in the liver. Therefore, most cancer patients do not manifest type B lactic acidosis and it is only in a few patients that this metabolic balance breaks down resulting in type B lactic acidosis. A number of mechanisms have been proposed for the development of type B lactic acidosis in the presence of malignancy and a few of these are the basis for potential therapeutic approaches.

Cancer patients with decreased hepatic clearance of lactate are thought to be at increased risk of developing lactic acidosis. This could occur in patients with extensive liver metastasis, although additional factors are likely to be involved because many cases reported in the literature have no laboratory or radiographic evidence of hepatic infiltration. This raises the possibility of a metabolic impairment of gluconeogenic pathways without a structural correlate as one of the mechanisms of lactic acidosis in cancer patients.

Sillos et al.[2] reviewed 53 cases published before 2001 of patients with leukemia or lymphoma and lactic acidosis, and also retrospectively analyzed three cases in which levels of insulin-like growth factor (IGF) (I and II) and tumor necrosis factor $\alpha$ were measured. His group proposed that the overproduction of lactate could be related to an increase in the glycolytic rate in tumor cells, secondary to an aberrant IGF signaling system that induces the overexpression of hexokinase type II, the rate-limiting enzyme involved in glucose consumption by the cell (glycolysis). This could also contribute to the development of hypoglycemia in patients with type B lactic acidosis.

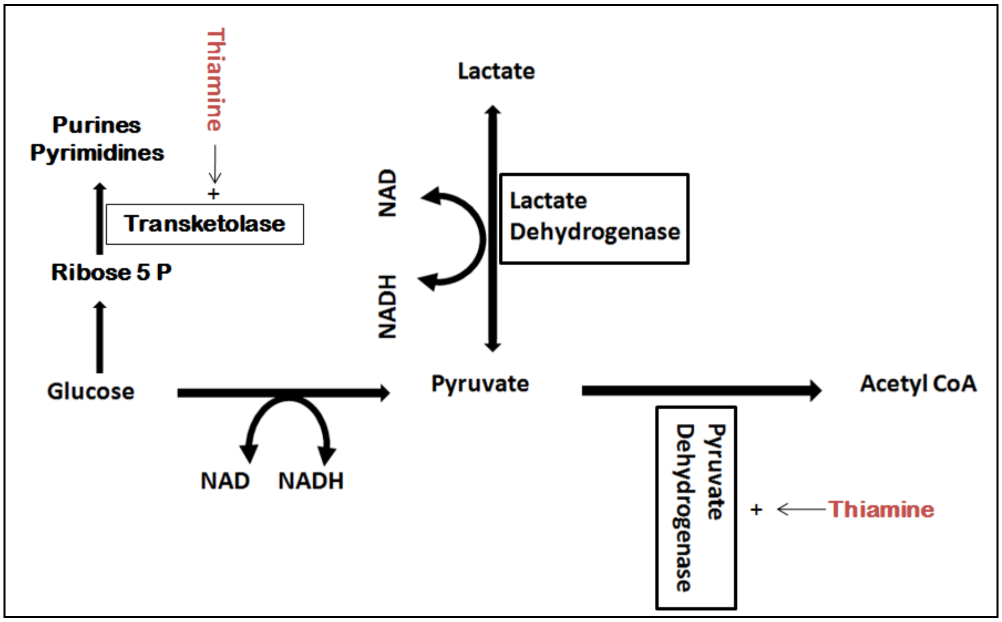

**FIGURE 2.** Lactic acid synthesis and role of thiamine as a cofactor of pyruvate dehydrogenase and transketolase enzyme systems.

Vitamin deficiencies have also been associated in the development of lactic acidosis[10,11,12,13]. In the absence of thiamine, pyruvate dehydrogenase is unable to convert pyruvate into acetyl-CoA, driving the accumulated pyruvate towards the production of lactate. In the case reported by Svahn et al.[13] of a patient with acute leukemia receiving total parenteral nutrition, improvement was achieved after the administration of thiamine supplementation. Unfortunately, erythrocyte transketolase activity was not measured prior to the treatment to confirm whether thiamine supplementation was indeed responsible for the patient's improvement.

Another possible mechanism is the embolization of malignant cells into the microvasculature, causing a state of hypoperfusion and therefore an increase of anaerobic metabolism[14].

## PROSPECTS OF THERAPY

Treatment options for type B lactic acidosis have not been fully established. It is not clear if there is a role for administering thiamine to patients who may have normal serum levels of the vitamin. The rationale for this would be to drive pyruvate towards the synthesis of acetyl-CoA instead of being shunted towards the production of lactic acid. On the other hand, there are reports that mention an increase in tumor growth when excess thiamine is given because it is also a cofactor of transketolase (Fig. 2), a rate-limiting enzyme of the pentose monophosphate shunt needed for the synthesis of ribose[15], which is a precursor of DNA. This would favor the synthesis of tumoral DNA. Clinical trials evaluating the role of thiamine in type B lactic acidosis secondary to cancer have not been performed.

Hemodialysis has also been proposed as a way of clearing the excess of lactic acid in patients with cancer. Prikis et al.[16] reported a case of a patient with undiagnosed B-cell lymphoma with severe lactic acidosis in which large amounts of bicarbonate were administered while SLED was performed. They suggest that this approach is a bridge before treating the patients for the underlying disorder, also reducing the need for mechanical ventilation and its accompanying complications. However, they acknowledge that alkali therapy alone is known to have several deleterious effects (decreased cardiac contractility, paradoxical production of intracellular lactic acid).

Treating the underlying malignancy with chemotherapy is the most widely used strategy. On the other hand, only a few patients with type B lactic acidosis seem to have transient improvement[13,17,18] and most patients are unaffected by it.

Finally, the outcome of the majority of patients that develop type B lactic acidosis secondary to malignancy is invariably fatal, suggesting that lactic acidosis may serve as a marker of poor prognosis.

Our patient received the three treatment strategies that have been tried in the cases reported. Although she had an adequate response to them, her outcome was unfavorable and she subsequently died, which is compatible with the poor prognosis that is reported in patients with cancer who develop type B lactic acidosis.

## CONCLUSION

Type B lactic acidosis is a rare complication of cancer, most frequently reported in hematologic malignancies. The proposed mechanisms by which it occurs include an aberrant IGF pathway, abnormal liver clearance of lactic acid from impaired gluconeogenesis with or without malignant infiltration, microembolization of malignant cells in the vasculature of the tumor, and thiamine deficiency. There are no randomized trials comparing treatment options, which include thiamine supplementation, renal replacement therapy with bicarbonate supplementation, and chemotherapy. Further studies are needed to evaluate the role of thiamine in the treatment of type B lactic acidosis secondary to malignancy, especially regarding its possible paradoxical increase in tumor growth that results from increased nucleic acid synthesis. Our patient showed a favorable response after undergoing hemodialysis, receiving thiamine, and a short course of intravenous isotonic bicarbonate infusion. She also received high-dose prednisone as an initial part of her chemotherapy. It is unclear if any or all interventions resulted in the transient improvement of the patient’s clinical status.

Although formal prospective trials are lacking, type B lactic acidosis in patients with cancer seems to be a marker of poor prognosis regardless of the treatment offered and may be invariably fatal. Future research should focus on potential therapy based on the pathogenic mechanisms that lead to type B lactic acidosis in cancer patients.
